# Progression of Kidney Disease in Non-Diabetic Patients with Coronary Artery Disease: Predictive Role of Circulating Matrix Metalloproteinase-2, -3, and -9

**DOI:** 10.1371/journal.pone.0070132

**Published:** 2013-07-26

**Authors:** Ta-Wei Hsu, Ko-Lin Kuo, Szu-Chun Hung, Po-Hsun Huang, Jaw-Wen Chen, Der-Cherng Tarng

**Affiliations:** 1 Division of Nephrology, National Yang-Ming University Hospital, Yilan, Taiwan; 2 Division of Nephrology, Buddhist Tzu Chi Hospital Taipei Branch, Taipei, Taiwan; 3 Division of Cardiology, Taipei Veterans General Hospital, Taipei, Taiwan; 4 Institute of Clinical Medicine, National Yang-Ming University, Taipei, Taiwan; 5 Division of Nephrology, Department of Medicine, Taipei Veterans General Hospital, Taipei, Taiwan; 6 Department and Institute of Physiology, National Yang-Ming University, Taipei, Taiwan; Universidade de Sao Paulo, Brazil

## Abstract

**Background:**

Circulating matrix metalloproteinase (MMP)-2, -3 and -9 are well recognized in predicting cardiovascular outcome in coronary artery disease (CAD), but their risks for chronic kidney disease (CKD) are lacking. Therefore, the present study aimed to investigate whether circulating MMP levels could independently predict future kidney disease progression in non-diabetic CAD patients.

**Methods:**

The prospective study enrolled 251 non-diabetic subjects referred for coronary angiography, containing normal coronary artery (n = 30) and CAD with insignificant (n = 95) and significant (n = 126) stenosis. Estimated glomerular filtration rate (eGFR) was calculated using the CKD-EPI formula. eGFR decline rate was calculated and the primary endpoint was a decline in eGFR over 25% from baseline.

**Results:**

The eGFR decline rate (ml/min/1.73 m^2^ per year) in patients with CAD (1.22 [−1.27, 1.05]) was greater than that in those with normal coronary artery (0.21 [−2.63, 0.47], *P*<0.01). The circulating MMP-2, -3 and -9 were independently associated with faster eGFR decline among CAD patients. The mean follow-up period was 8.5±2.4 years, and 39 patients reached the primary endpoint. In multivariate Cox regression model, the adjusted hazard ratios of MMP-2 ≥861 ng/mL, MMP-3 ≥227 ng/mL and MMP-9 ≥49 ng/mL for predicting CKD progression were 2.47 (95% CI, 1.21 to 5.07), 2.15 (1.12 to 4.18), and 4.71 (2.14 to 10.4), respectively. While added to a model of conventional risk factors and baseline eGFR, MMP-2, -3 and -9 further significantly improved the model predictability for CKD progression (c statistic, 0.817). In the sensitivity analyses, the results were similar no matter if we changed the endpoints of a decline of >20% in eGFR from baseline or final eGFR < 60 mL/min/1.73 m^2^.

**Conclusion:**

Circulating MMP-2, -3 and -9 are independently associated with kidney disease progression in non-diabetic CAD patients and add incremental predictive power to conventional risk factors.

## Introduction

Matrix metalloproteinases (MMPs) form a family of zinc-dependent enzymes with proteolytic activity against connective tissue proteins [1]. Increased expression and activity of MMPs have been identified in various pathological processes, such as atherosclerosis, nephritis, arthritis, cancer, tissue ulcers, and fibrosis [2]. MMP-2, -3, and -9 secreted by macrophages and other inflammatory cells accelerate atherosclerotic progression and destabilize vulnerable plaque in animal models [3]. Moreover, over-expressions of MMP-2, -3, and -9 are found in human atherosclerotic plaque [3], and their plasma levels have been identified as a novel predictor of long-term prognosis and cardiovascular mortality in coronary artery disease (CAD) patients [4]–[7]. In the kidney, MMP-2, -3, and -9 are expressed predominantly in mesangial cells and podocytes of glomeruli and in the tubular epithelial cells, mainly in the proximal tubules. Recent data proposed a role for MMPs in the pathophysiology of both acute kidney injury and chronic kidney disease (CKD) [1], [8]. Circulating levels of MMPs presumably reflect their expression in the whole body as well as the kidney. In a small study, MMP-2 and -9 levels in the serum of 10 patients with glomerulonephritis paralleled their expressions in kidney biopsy tissues [9]. In the cross-sectional studies, plasma MMP-2 levels were positively correlated with serum creatinine levels in CKD patients [10], [11]. Furthermore, it has been shown that increased plasma level of MMP-9 predicted the appearance of microalbuminuria in noninsulin-dependent diabetes mellitus [12]. Till now, to our best knowledge, the relationship between plasma MMP-2, -3 and -9 levels and kidney disease progression is unclear in CAD patients.

The nexus between CAD and CKD is extremely complex and partially attributable to some risk factors in CAD. Diabetes mellitus and proteinuria have been known as important factors for CKD progression. Therefore, we focus on a CAD cohort without diabetes and proteinuria to appraise whether the estimated glomerular filtration rate (eGFR) decline rate in CAD is greater than that of subjects with normal coronary artery, and to assess whether circulating MMP-2, -3, and -9 levels at baseline could independently predict future progression of kidney disease and overall mortality.

## Materials and Methods

### Ethics Statement

The study complied with the Declaration of Helsinki and was approved by the institutional review board of Taipei Veterans General Hospital. All participants gave their written informed consent before inclusion.

### Study Patients

Between March 1999 and June 2003, 601 consecutive non-diabetic patients who were referred to the Catheterization Center of Taipei Veterans General Hospital for coronary angiography by reason of angina and/or suspected CAD. Patients were excluded if they had acute coronary syndrome including acute myocardial infarction or unstable angina during hospitalization, uncontrolled congestive heart failure, unstable hemodynamic status, chronic liver disease, renal insufficiency (serum creatinine >1.5 mg/dL for males and >1.2 mg/dL for females), proteinuria by dipstick, chronic systemic inflammatory disease, and malignancy with expected life span less than 1 year. Initially, 346 consecutive patients were enrolled in the study. Two expert angiographers, who were unaware of the patient’s clinical and laboratory data, independently reviewed the coronary angiography. Accordingly, 174 patients had significant CAD (the presence of ≥50% stenosis in at least one major coronary artery), 125 had insignificant CAD (the presence of <50% but ≥20% stenosis in at least one major coronary artery), and 47 had normal coronary artery (<20% stenosis at all major coronary arteries). Baseline demographic data were recorded at the time of recruitment. All patients were enrolled by one physician to minimize interobserver variations. The protocol was approved by the Committee on Human Research at Taipei Veterans General Hospital, and informed consent was obtained from each participant.

### Laboratory Measurements

A 20-mL venous blood was drawn in the early morning after overnight fasting in each patient before coronary angiogram. The plasma and serum samples were stored at −80°C until use. Biochemical parameters including total cholesterol, triglyceride, low-density lipoprotein (LDL) and high-density lipoprotein (HDL) cholesterol, glucose, albumin, creatinine, calcium, phosphate, and uric acid were determined using commercial kits by a Hitachi 7600 autoanalyzer (Roche Modular®; Hitachi Ltd, Tokyo, Japan). Serum high-sensitivity CRP (hs-CRP) was measured by an immunoturbidimetric assay, using rate nephrelometry (IMMAGE®; Beckman Coulter, Galway, Ireland).

Total form of MMP-2, -3, and -9 levels in plasma were determined using commercially available kits by enzyme-linked immunosorbent assay (Amersham Biosciences, Uppsala, Sweden) according to the manufacturer’s instructions. Within- and between-assay coefficients of variation determined in our laboratory were 5.4% and 8.2% for MMP-2, 2.4% and 9.8% for MMP-3, and 5.3% and 9.1% for MMP-9, respectively. The methodology in our study has been published before [5], [6].

### Assessment of Annual Decline Rate in eGFR

The eGFR was calculated using the CKD Epidemiology Collaboration (CKD-EPI) study equation [13]. The rate of annual decline in eGFR over the course of the study was determined from the slope of the plot of all outpatient eGFR measurements (median 6 measurements, range of 3 to 54 measurements) for each individual. At least three eGFR measurements were required to estimate the eGFR slope, which was calculated by linear regression analysis and expressed as ml/min/1.73 m^2^ per year.

### Prospective Follow-Up

After the baseline investigation, patients were followed prospectively until the study endpoints or the observation period was reached. The study was ended on December 31, 2009. There are two endpoints in this study. The primary endpoint was two consequent outpatient eGFR values that were more than 25% decline from baseline. The time reaching renal endpoint was the time to the first occurrence of a >25% decline in eGFR since enrollment. GFR decline rate of 1 ml/min/1.73 m^2^ per year over the age of 40 is suggested by classic inulin clearance study [14]. Mean eGFR in our patients was 74±15 ml/min/1.73 m^2^ at baseline. During a follow-up period of 8.5 years, a 25% decline of eGFR from baseline was 18 ml/min/1.73 m^2^ and exceeded the decline rate attributed to the aging process in the present study. The secondary endpoint was all-cause death. The cause of death was obtained according to the Taiwan National Death Registry. Among all participants, 95 patients were lost to follow-up with the observation time of <12 months and/or less than 3 outpatient eGFR measurements during the follow-up. They were further excluded from the study to avoid an incomplete observation in renal function change. Before starting the study, a sample size calculation was performed in which the statistical power was 80% and the 10-year kidney disease progression rate was estimated as 20% to 30% for the entire population. Inter-group difference would be reflected by a hazard ratio of at least 2.0. The required sample size was estimated between 70 and 100 patients. Therefore, a total of 221 CAD patients recruited in this study fulfilled the minimal requirement of case number.

### Sensitivity Analyses

To assess the reliability of our findings and minimize misclassification bias, we conducted the secondary analyses using different study endpoints of a decline of >20% in eGFR from baseline or final eGFR <60 mL/min/1.73 m^2^.

### Statistical Analysis

Descriptive statistics included means ± SD for continuous data and percentages for categorical data. The values of serum hs-CRP, MMP-2, -3, -9 and eGFR slope were not normally distributed and were reported as median with interquartile range (IQR). Potential differences between the two patient groups were assessed by the student’s *t* test for normally distributed data, the Mann-Whitney U test for non-normally distributed data, or the Pearson *x* ^2^ test for categorical variables. Linear regression analysis was used to identify the MMPs and other factors associated with eGFR slope. Because adjustment of baseline eGFR may disregard the notion that individuals with a rapid decline in eGFR in the past may have lower levels of eGFR at enrollment than those without [15]. We, therefore, excluded baseline eGFR from the linear regression model. The Kaplan-Meier technique (log-rank test) was applied to survival analysis. For the renal endpoint, observations were censored at the end of the study or the date that patients died, whichever occurred first. Cox proportional hazards model was used to examine the association of baseline variables with progression to renal endpoint and all-cause mortality. The multivariate Cox regression analysis was performed to evaluate the independent contribution of MMP levels to the risk of kidney disease progression and death, adjusting for age, gender, significant variables in univariate analysis, as well as established risk factors for a poor outcome. A backward elimination procedure was performed using *P*>0.05 to remove any identified independent predictors for kidney disease progression or death. To assess discrimination ability, *c* statistic for model of conventional risk factors, including age, sex, smoking status, BMI, systolic blood pressure, fasting glucose, total cholesterol, and eGFR (base model), then with stepwise addition of MMP-2, -3 and -9 values (newer biomarker model) was calculated for prediction of progression of CKD at 8 years of the study [16]. A *P-*value less than 0.05 was considered statistically significant. All statistical analyses were performed using the computer software Statistical Package of Social Science, version 16.0 (SPSS Inc., Chicago, IL).

## Results

Ninety five (27.5%) patients were excluded due to incomplete follow-up. Finally, a total of 251 (72.5%) patients from the baseline cohort could be assessed during the follow-up. Both groups did not differ significantly with respect to the demographic data and baseline laboratory parameters (Table S1 in File S1).

### Baseline Characteristics

The clinical characteristics of study subjects are summarized in Table 1. All CAD patients were stratified into two groups by the medians of baseline plasma MMP-2, -3, and -9 levels. Medians of MMP-2, MMP-3 and MMP-9 levels were 861, 227, and 49 ng/mL, respectively. Participants with baseline MMP-2, -3, and -9 higher than the medians had a significantly older age and a lower baseline eGFR, and those with MMP-9 and MMP-2 higher than the medians had a lower serum albumin and a higher serum hs-CRP, respectively.

**Table 1:** Demographic characteristics and laboratory data at baselines among CAD patients stratified by medians of matrix metalloproteinase-2, -3 and -9 levels, respectively.

Parameters	MMP-2 (ng/mL)		MMP-3 (ng/mL)		MMP-9 (ng/mL)	
	<861	≥861	<227	≥227	<49	≥49
Age (years)	65±12	69±8^b^	65±11	69±10^b^	65±11	71±7^b^
Male gender (%)	82.2	90.1	83.2	88.5	83.0	88.7
Hypertension (%)	74.5	69.5	70.6	73.6	72.3	71.0
Smoking history (%)	27.8	29.6	30.1	26.9	30.8	25.0
Body mass index (kg/m^2^)	25.6±3.5	25.2±3.4	25.6±3.6	25.2±3.2	25.6±3.5	25.0±3.2
Baseline GFR (mL/min per 1.73 m^2^)	75.9±16.5	71.3±14.0^a^	77.0±15.3	69.2±14.6^b^	75.4±15.7	69.9±14.4^a^
Fasting glucose (mg/dL)	97±20	96±13	97±14	96±19	97±18	94±12
Lipid profile						
Triglyceride (mg/dL)	136±58	125±70	135±60	124±70	145±58	120±87
Total cholesterol (mg/dL)	187±32	181±34	186±31	182±36	189±32	180±33
LDL-cholesterol (mg/dL)	119±28	115±31	118±28	116±31	120±29	114±30
HDL-cholesterol (mg/dL)	40±9	41±11	41±9	40±11	40±9	42±12
Serum albumin (g/dL)	4.11±032	4.07±0.31	4.08±030	4.09±0.34	4.13±029	4.02±0.32^a^
Calcium (mg/dL)	8.9±0.5	8.9±0.5	8.9±0.5	8.9±0.4	8.9±0.5	8.9±0.4
Phosphate (mg/dL)	3.3±0.5	3.3±0.6	3.3±0.6	3.4±0.6	3.3±0.6	3.3±0.5
Uric acid (mg/dL)	6.8±2.1	7.2±1.9	6.8±1.8	7.3±2.2	7.0±2.1	7.0±1.8
High-sensitivity CRP (mg/L)	0.69	1.03^a^	0.85	0.90	0.72	1.00
	[0.34 to 1.55]	[0.48 to 3.06]	[0.48 to 2.38]	[0.36 to 2.45]	[0.38 to 2.18]	[0.42 to 2.65]
Medications at enrollment						
Antiplatelet agents (%)	80.0	78.3	82.4	76.0	84.4	74.2
Nitrate (%)	35.5	34.1	31.6	37.3	33.3	32.1
Calcium channel blockers						
DHP (%)	20.0	18.4	18.4	20.2	18.2	22.2
Non-DHP (%)	35.5	34.1	31.6	37.3	33.3	32.1
β blockers (%)	20.9	14.9	21.6	13.3	21.4	14.8
ACEI and/or ARB (%)	41.8	45.0	44.0	42.7	42.9	45.2
Statins (%)	23.6	18.4	22.8	20.2	21.4	21.9

Medians of baseline plasma MMP-2, MMP-3, and MMP-9 levels were 861 ng/mL, 227 ng/mL, and 49 ng/mL, respectively.

Comparison between two groups of patients with different MMP-2, -3, and -9 levels by the student’s *t* test, Pearson *x* ^2^ test, or the Mann-Whitney U test, as appropriate.

a *P*<0.05;

b *P*<0.01.

ACEI, angiotensin-converting enzyme inhibitors; ARB, angiotensin II receptor blockers; CAD, coronary artery disease; CRP, C-reactive protein; DHP, dihydropyridine; eGFR, estimated glomerular filtration rate; HDL, high density lipoprotein; LDL, low density lipoprotein; MMP, matrix metalloproteinase.

### Baseline MMP Levels Associated with the Decline Rate of eGFR

**Figure 1:**
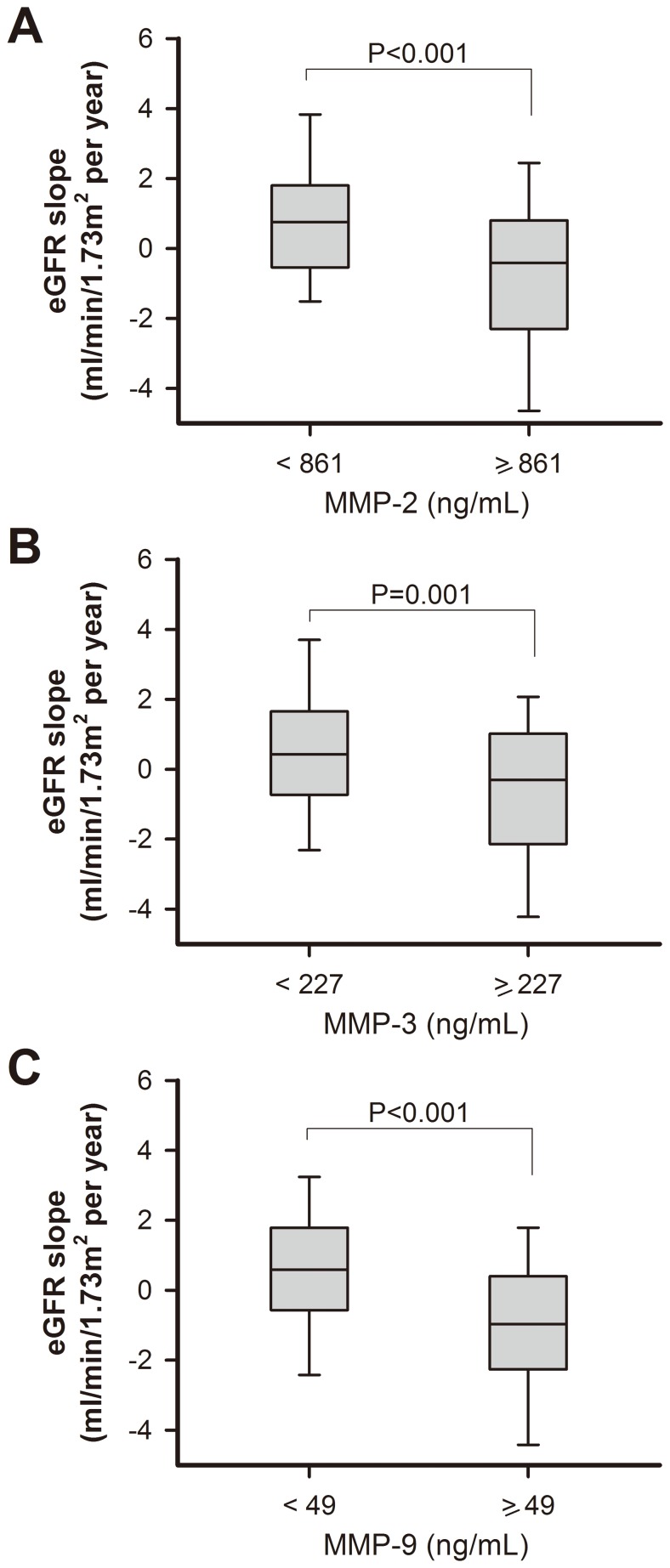
Whisker plots showing the 10th, 25th, 50th, 75th and 90th percentiles distribution of eGFR slope. Difference between patients with MMP-2 (A), MMP-3 (B) and MMP-9 (C) above and below the medians, respectively. Medians of baseline plasma MMP-2, MMP-3 and MMP-9 levels were 861 ng/mL, 227 ng/mL, and 49 ng/mL, respectively. Abbreviations: eGFR, estimated glomerular filtration rate; MMP, matrix metalloproteinase.

Mean decline rate of eGFR (ml/min/1.73 m^2^ per year) in patients with significant CAD (1.22 [−1.27, 1.05]) was greater than that in those with normal coronary artery (0.21 [−2.63, 0.47], *P*<0.01). In multivariate regression analysis among CAD patients, the independent determinants of annual eGFR decline rate were age (*r = –*0.20, *P*<0.01), serum levels of albumin (*r* = 0.16, *P* = 0.02) and hs-CRP (*r = –*0.14, *P* = 0.04), and plasma levels of MMP-2 (*r = –*0.22, *P*<0.01), MMP-3 (*r = –*0.19, *P* = 0.01), and MMP-9 (*r = –*0.29, *P*<0.01). Furthermore, using baseline MMP-2, MMP-3 or MMP-9 levels above *versus* below the respective medians as categorical variables (Figure 1), we found that patients with baseline MMP-2, -3, and -9 higher than the medians had a significantly greater annual decline rate in eGFR as compared to those with plasma levels lower than the medians (*P*<0.001 for MMP-2, *P*<0.01 for MMP-3, and *P*<0.001 for MMP-9, respectively).

### Baseline MMP Levels Predicted the Kidney Disease Progression

During a mean follow-up period of 8.5±2.4 years, 39 patients reached the renal endpoint of an eGFR decline >25% from baseline. Compared with the non-progressors (Table 2), progressors significantly had older age; lower levels of basal eGFR, triglyceride and albumin; and higher levels of hs-CRP and plasma MMP-2, -3, and -9 at baseline.

**Table 2:** Comparison of baseline parameters between patients who did and did not reach the endpoint defined by an eGFR decline more than 25% from baseline.

	With Renal Endpoint	Without Renal Endpoint	
Parameters	(n = 39)	(n = 182)	*P* value^a^
Age (years)	71.9±7.9	65.9±10.9	**<0.01**
Male gender (%)	89.7	84.6	0.41
Hypertension (%)	81.1	69.3	0.15
Smoking history (%)	24.3	28.7	0.59
Body mass index (kg/m^2^)	24.9±3.4	25.5±3.4	0.31
Baseline eGFR (mL/min per 1.73 m^2^)	66.2±13.4	75.1±15.4	**<0.01**
Fasting glucose (mg/dL)	95.3±13.0	96.7±17.2	0.64
Lipid profile			
Triglyceride (mg/dL)	106±59	136±65	**0.01**
Total cholesterol (mg/dL)	179±31	185±34	0.31
LDL-cholesterol (mg/dL)	116±28	118±30	0.76
HDL-cholesterol (mg/dL)	42±12	40±10	0.25
Serum albumin (g/dL)	3.98±0.38	4.11±0.29	**0.02**
Calcium (mg/dL)	8.8±0.5	8.9±0.5	0.19
Phosphate (mg/dL)	3.3±0.5	3.3±0.6	0.91
Uric acid (mg/dL)	7.1±1.9	7.0±2.0	0.83
High-sensitivity CRP (mg/L)	1.59 [0.69 to 4.18]	0.72 [0.37 to 2.08]	**<0.01**
Matrix metalloproteinase-2 (ng/mL)	951 [859 to 1085]	883 [728 to 1012]	**0.04**
Matrix metalloproteinase-3 (ng/mL)	254 [204 to 337]	208 [167 to 260]	**<0.01**
Matrix metalloproteinase-9 (ng/mL)	54 [43 to 71]	42 [35 to 53]	**<0.01**
Medications at enrollment			
Antiplatelet agents (%)	74.3	72.5	0.82
Nitrate (%)	38.5	42.3	0.66
Calcium channel blockers: DHP (%)	20.5	20.8	0.95
Calcium channel blockers: Non-DHP (%)	43.6	39.6	0.64
β blockers (%)	23.3	43.9	0.22
ACEI and/or ARB use (%)	56.4	56.0	0.98
Statins (%)	17.9	25.2	0.33

Comparison between two groups of patients by the student’s *t* test, Pearson *x* ^2^ test, or Mann-Whitney U test, as appropriate.

ACEI, angiotensin-converting enzyme inhibitors; ARB, angiotensin II receptor blockers; CAD, coronary artery disease; CRP, C-reactive protein; DHP, dihydropyridine; eGFR, estimated glomerular filtration rate; HDL, high density lipoprotein; LDL, low density lipoprotein.

**Figure 2:**
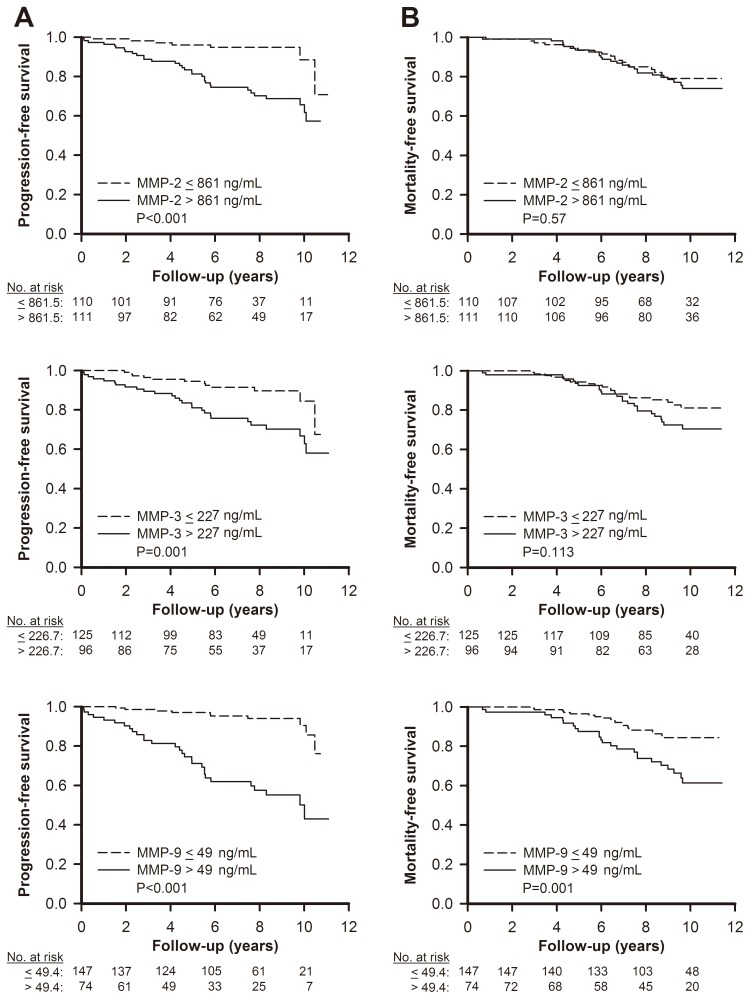
Kaplan-Meier analysis of survival curves in CAD patients. Progression-free (A) and mortality-free (B) survivals are the study endpoints. Difference between patients with MMP-2, MMP-3 and MMP-9 above and below the medians, respectively, by log-rank test. Medians of baseline plasma MMP-2, MMP-3 and MMP-9 levels were 861 ng/mL, 227 ng/mL, and 49 ng/mL, respectively. Abbreviations: CAD, coronary artery disease; MMP, matrix metalloproteinase.

Using Kaplan-Meier analysis, patients with baseline plasma MMP-2, -3, and -9 above the respective medians had significant higher risks for progression to renal endpoint than those with plasma levels below the medians (Log-rank test *P*<0.01 for MMP-2, *P*<0.01 for MMP-3, and *P*<0.01 for MMP-9, respectively, Figure 2A). The mortality rate was 28.4% and 26 deaths (40%) were CV-related, while 39 deaths (60%) were non-CV-related. Only patients with baseline plasma MMP-9 level ≥49 ng/mL had a significantly higher risk for death (Log-rank test *P* = 0.001; Figure 2B).

Univariate Cox regression analysis (Table 3) showed that the predictors of progression to renal endpoint included age, basal eGFR, serum albumin and hs-CRP, and plasma MMP-2 (≥861 ng/mL), MMP-3 (≥227 ng/mL) and MMP-9 (≥49 ng/mL) at baseline. Meanwhile, the predictors of mortality were age, basal eGFR, serum albumin, plasma MMP-9 (≥49 ng/mL), and use of angiotensin-converting enzyme inhibitors (ACEI) and/or angiotensin II receptor blockers (ARB).

**Table 3:** Univariate analysis for the predictors of kidney disease progression and mortality in patients with coronary artery disease.

	Kidney Disease Progression		Mortality	
Variables	Hazard Ratio, 95% CI	*P* value	Hazard Ratio, 95% CI	*P* value
Age: per 10 years	2.09 [1.36 to 3.23]	**<0.01**	1.66 [1.22 to 2.25]	**<0.01**
Gender: male vs. female	1.43 [0.51 to 4.03]	0.49	4.04 [1.27 to 12.9]	**0.02**
Hypertension: yes vs. no	1.62 [0.71 to 3.68]	0.25	1.34 [0.44 to 1.89]	0.45
Smoking: ever vs. never	1.74 [0.85 to 2.57]	0.63	1.87 [0.65 to 2.88]	0.73
Body mass index: per 1 kg/m^2^	0.94 [0.84 to 1.04]	0.22	0.95 [0.88 to 1.04]	0.27
CAD: multiple vessels vs. single vessel	1.94 [0.39 to 4.01]	0.07	2.14 [1.23 to 3.71]	**<0.01**
Baseline eGFR: per 10 mL/min per 1.73 m^2^	0.68 [0.54 to 0.85]	**<0.01**	0.72 [0.61 to 0.86]	**<0.01**
Fasting glucose: per 1 mg/dL	0.99 [0.97 to 1.01]	0.51	1.00 [0.99 to 1.02]	0.70
Triglyceride: per 1 mg/dL	0.99 [0.98 to 1.29]	0.61	0.99 [0.99 to 1.00]	0.28
Total cholesterol: per 1 mg/dL	0.99 [0.98 to 1.00]	0.09	0.99 [0.98 to 0.99]	0.05
LDL-cholesterol: per 1 mg/dL	0.99 [0.98 to 1.01]	0.44	0.99 [0.98 to 1.01]	0.17
HDL-cholesterol: per 1 mg/dL	1.01 [0.98 to 1.04]	0.34	0.98 [0.96 to 1.01]	0.31
Serum albumin: per 1.0 g/dL	0.83 [0.67 to 0.91]	**0.03**	0.86 [0.73 to 0.97]	**0.04**
Calcium: per 1 mg/dL	0.57 [0.33 to 1.01]	0.51	0.69 [0.43 to 1.09]	0.11
Phosphate: per 1 mg/dL	0.89 [0.50 to 1.57]	0.68	1.25 [0.82 to 1.91]	0.29
Uric acid: per 1 mg/dL	1.01 [0.86 to 1.18]	0.91	1.02 [0.90 to 1.15]	0.79
High-sensitivity CRP: per 1.0 mg/L	1.06 [1.01 to 1.29]	**0.04**	1.76 [0.96 to 3.21]	0.07
MMP-2≥861 ng/mL	4.58 [2.02 to 10.4]	**<0.01**	1.25 [0.68 to 2.29]	0.47
MMP-3≥227 ng/mL	2.89 [1.46 to 5.71]	**<0.01**	1.56 [0.86 to 2.84]	0.15
MMP-9≥49 ng/mL	7.58 [3.69 to 15.6]	**<0.01**	2.76 [1.51 to 5.05]	**<0.01**
Medications				
Antiplatelet agent use	1.11 [0.54 to 2.29]	0.76	1.15 [0.66 to 1.99]	0.63
Nitrate use	0.91 [0.48 to 1.73]	0.77	1.03 [0.98 to 2.73]	0.69
Calcium channel blockers: DHP use	0.84 [0.38 to 1.84]	0.67	0.75 [0.39 to 1.43]	0.38
Calcium channel blockers: Non-DPH use	1.19 [0.63 to 2.25]	0.59	1.04 [0.63 to 1.70]	0.89
β blocker use	0.69 [0.36 to 1.35]	0.28	0.77 [0.47 to 1.29]	0.33
ACEI and/or ARB use	0.97 [0.51 to 1.84]	0.93	0.53 [0.34 to 0.92]	**0.02**
Statin use	0.60 [0.27 to 1.37]	0.23	1.02 [0.58 to 1.80]	0.94

ACEI, angiotensin-converting enzyme inhibitors; ARB, angiotensin II receptor blockers; CAD, coronary artery disease; CI, confidence interval; CRP, C-reactive protein; DHP, dihydropyridine; eGFR, estimated glomerular filtration rate; HDL, high density lipoprotein; LDL, low density lipoprotein; MMP, matrix metalloproteinase.

In multivariate Cox proportional hazard model (Table 4), only baseline MMP-9 (*P* = 0.03) along with age (*P* = 0.03) and basal eGFR (*P* = 0.04) were the significantly risk factors for mortality during the follow-up. In contrast, the adjusted hazard ratios of MMP-2≥861 ng/mL, MMP-3≥227 ng/mL and MMP-9≥49 ng/mL for predicting CKD progression were 2.47 (95% CI, 1.21 to 5.07), 2.15 (95% CI, 1.12 to 4.18), and 4.71 (95% CI, 2.14 to 10.4). In the sensitivity analyses, the results were similar no matter if we changed the study endpoints (Table S2 in File S1).

**Table 4:** Multivariate Cox proportional hazard regression models for the prediction of kidney disease progression and mortality among patients with coronary artery disease.

Variables	Hazard Ratio	95% CI	*P* value
**Kidney disease progression**			
eGFR: per 10 mL/min per1.73 m^2^ increase	0.86	[0.68 to 0.91]	0.03
MMP-2≥861 ng/mL	2.47	[1.21 to 5.07]	0.01
MMP-3≥227 ng/mL	2.15	[1.12 to 4.18]	0.02
MMP-9≥49 ng/mL	4.71	[2.14 to 10.4]	**<**0.01
**Overall mortality**			
Age: per 10 years increase	1.31	[1.10 to 2.67]	0.03
eGFR: per 10 mL/min per1.73 m^2^ increase	0.75	[0.60 to 0.93]	0.02
MMP-9≥49 ng/mL	2.25	[1.22 to 4.14]	0.01

Medians of baseline plasma MMP-2, MMP-3 and MMP-9 levels were 861 ng/mL, 227 ng/mL, and 49 ng/mL, respectively.

CI, confidence interval; eGFR, estimated glomerular filtration rate; MMP, matrix metalloproteinase.

### Baseline MMP Levels Significantly Increased the Predictability for Kidney Disease Progression

**Figure 3:**
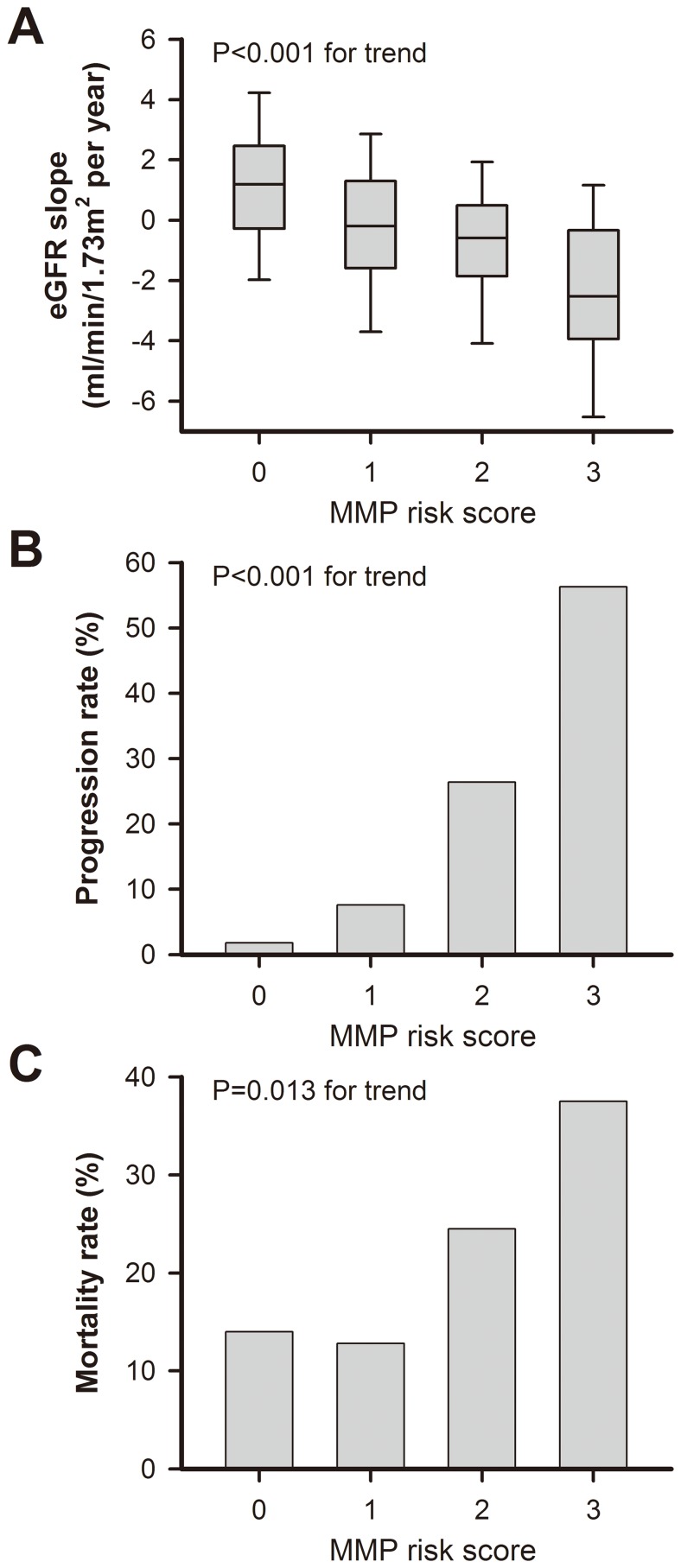
MMP risk score in renal and mortality outcomes in patients with stable CAD. Outcomes contain eGFR decline rate (A), incidence of renal progression (B), and overall mortality rate (C). Abbreviations: CAD, coronary artery disease; eGFR, estimated glomerular filtration rate; MMP, matrix metalloproteinase.

To test discrimination ability, plasma MMP-2, -3 and -9 levels at baseline were incorporated into the conventional risk factors (base model) for predicting CKD progression (Table 5). The *c* statistic of base model was 0.719. Addition of MMP-2 plus MMP-9 and three MMP values to conventional risk factors (newer marker model) further improved the model predictability with increment in the c statistic of 0.069 and 0.102 (*P*<0.05), respectively. To assess the clinical implications of MMPs in CAD patients, we further developed a risk score with a range of zero to three by using the baseline MMP levels. A score of zero was defined when the plasma levels of three MMPs were all below the medians. Scores of one, two and three were defined as one, two or all of the three MMPs being above the corresponding median values, respectively. As shown in Figure 3, the higher the MMP risk score, the faster the eGFR decline rate (*P*<0.001), the higher the incidence of kidney disease progression (*P*<0.001), and the higher the mortality rate (*P* = 0.01).

**Table 5:** Discriminative ability of conventional risk factors and addition of MMPs for prediction of chronic kidney disease progression.

Risk factors and biomarkers	*c* statistic^a^	Change in *c* statistic^b^		*P* c
	(95% CI)			
Conventional risk factors only	0.719 (0.641 to 0.788)	–	referent	
+ MMP-2	0.725 (0.649 to 0.789)	0.009(–0.031 to 0.069)	0.47	
+ MMP-3	0.722 (0.645 to 0.788)	0.005(–0.036 to 0.056)	0.48	
+ MMP-9	0.744 (0.700 to 0.817)	0.017(–0.021 to 0.115)	0.15	
+ MMP-2 + MMP-3	0.754 (0.699 to 0.803)	0.056(–0.019 to 0.133)	0.15	
+ MMP-3 + MMP-9	0.745 (0.669 to 0.811)	0.028(–0.018 to 0.074)	0.24	
+ MMP-2 + MMP-9	0.797 (0.703 to 0.840)	0.069 (0.030 to 0.145)	**0.04**	
+ MMP-2 + MMP-3+ MMP-9	0.817 (0.721 to 0.889)	0.102 (0.081 to 0.176)	**0.03**	

a Risk prediction was assessed by the *c* statistic. Each newer marker was stepwise added to model of conventional risk factors (base model) to assess the *c* statistic for predicting progression of chronic kidney disease at 8 years. Conventional risk factors included age, sex, smoking status, systolic blood pressure, body mass index, fasting glucose, total cholesterol, and baseline eGFR.

b Increment in the *c* statistic in the newer marker model versus the base model. The *c* statistic is corrected for overoptimism by using 100 bootstrap repetitions.

c A *P* value <0.05 indicated that change in *c* statistic in the newer marker model as compared to the base model is statistically significant.

CI, confidence interval; eGFR, estimated glomerular filtration rate; MMP, matrix metalloproteinase.

## Discussion

This study prospectively demonstrated that baseline MMP-2, -3 and -9 levels were the independent predictors for faster eGFR decline and subsequent kidney disease progression (Table 4). Moreover, baseline plasma MMP-9 was independently associated with an increased risk of mortality. We also provided novel evidence that combining the MMP-2, -3 and -9 significantly adds the predictive value of conventional risk factors for kidney disease progression in a non-diabetic CAD cohort.

Long-term outcomes of CAD, such as major adverse cardiovascular events and all-cause mortality, are well documented. However, the impact of CAD on renal outcomes like progression of kidney disease is still largely undefined. Elsayed *et al.* [17] found that cardiovascular disease is an independent risk factor for initiation and progression of CKD, but the specific types of cardiovascular disease were not discriminated. Several studies reported that atherosclerosis at non-renal sites is associated with renal function decline [18]–[20]. Kiyosue *et al.* [21] recently showed that the severity of CAD is correlated with the worsening of renal function. In the same line, our findings further indicated that among individuals referred for coronary angiography, non-diabetic patients with notably stenotic CAD had significantly greater decline in eGFR compared with normal coronary artery by rate of 1.1 mL/min/1.73 m^2^ per year.

Guidelines for the assessment of the risk of a faster eGFR decline or kidney disease progression remain focused largely on conventional risk factors [22]. In the present study, the most compelling finding is that elevated MMP-2,-3 and -9 plasma levels are prognostic biomarkers for kidney disease progression independent of the conventional risk factors. The other significant prognostic factor is low eGFR at baseline. Although the prediction of progression by MMPs might be an effect of residual confounding by severity of kidney function, we have confirmed that the associations between high MMP expression or low eGFR with kidney disease progression are independent of each other (Table 4). Similar findings by Ebihara et al.^12^ have shown that plasma MMP-9 concentration predicted the development of microalbuminuria in type 2 diabetes by an observation period of 4 years.

The role of MMPs in renal fibrosis is probably dual during the different phases of the pathology evolution [23]. MMPs have the potential to fragment some extracellular matrix for removal, but also cleave nonmatrix substrates, releasing profibrotic growth factors that paradoxically trigger unwelcome consequences [24]. Compelling evidence has shown that MMP-9 and MMP-2 might promote renal fibrogenesis by stimulating epithelial-mesenchymal transition. MMP-9 production in podocytes can be modulated by high glucose, albumin and transforming growth factor (TGF)-β [25]–[27]. Podocyte binds to glomerular basement membrane by integrins. Elevated integrin-linked kinase induced by MMP-9 causes podocyte detachment from glomerular basement membrane and podocyturia *in vivo* [28]. Overexpression of MMP-2 in transgenic mice promotes renal fibrosis and generates the entire spectrum of pathological and functional changed characteristics mimicking human CKD [29], [30]. Since MMPs are of relatively large size that precludes their filtration and excretion in the urine, the circulating levels may reflect their expressions in the diseased kidney as well as atherosclerotic plaque. The hypothesis is substantiated by the findings that serum MMP-2 and -9 levels paralleled their expressions in kidney biopsy tissues of 10 patients with glomerulonephritis [9]. Taken together, it is implicated that the increased MMP-2 and -9 levels might reflect a profibrotic activity in kidneys to accelerate disease progression, rather than a compensatory mechanism trying to limit the ongoing extracellular matrix accumulation.

Individual MMP modestly provided incremental benefit in risk prediction of kidney disease progression when added separately to a base model, but combining the three MMPs offered the greatest improvement in risk prediction beyond conventional risk factors in our study (Table 5). This notion is biologically plausible because the overlapping activity and specificity of MMPs make it difficult to dissect individual actions *in vivo.* Most MMPs are secreted as proMMPs and activation usually requires cleavage of the prodomain by plasmin or other MMPs. MMP-2 and MMP-3 activate pro-MMP-9 by cleaving their prodomains [31]–[34]. Several MMPs are able to release growth factors by cleaving either the growth-factor binding protein or shedding of membrane-bound cytokines from cell surface. Proteolytic activation of cytokines or growth factors by one MMP might lead to upregulation of other MMPs. Furthermore, a number of the MMP promoters share common structural features and, therefore are co-regulated in their expression to some extent [35].

In our statistical analysis, older age, low basal eGFR and higher MMP-9 levels at baseline were the independent predictors of mortality in CAD patients over a mean follow up of 8.5 years. These findings are in general agreement with our previous study [6] and other reports [7], [36], which have analyzed risk factors and causes of death among CAD patients.

Some limitations of this study should be acknowledged. First, the number of serum creatinine measurements for measuring the eGFR slope varied in each patient, and thus, the calculation of eGFR slope was not uniform in every subject. However, to avoid an unreliable estimation of eGFR slope, we excluded 95 patients with either fewer than three eGFR measurements or a follow-up period of <12 months from the study. Second, the predominant sources of MMPs detected in the circulation are unknown. Circulating levels of MMPs presumably reflect their expression in the whole body as well as in the kidney. Elevation of MMPs could also result from impaired filtration in the damaged kidneys, but this seems unlikely since the relatively large size of MMPs (∼60 kDa) would apparently preclude their excretion from the kidneys. Finally, some medications have been shown to alter the expression of MMPs in CAD patients [9], [37], [38]. Nevertheless, in multivariate Cox proportional hazards model, no evidence indicated that medications such as statins, nitrates and ACEI/ARB modified the risk for progression to renal endpoint and mortality in our patients.

In summary, circulating MMP-2, -3 and -9 are independently associated with subsequent progression of kidney disease in non-diabetic CAD patients. Plasma MMP-2,-3 and -9 provide information that is complementary to conventional risk factors and baseline eGFR for kidney disease progression. The potential impact of these results, if confirmed and expanded upon in future studies, includes providing new insight into mechanisms of CKD and the possibility of improving screening, intervention, and prevention.

## Supporting Information

File S1 **Includes Table S1 and S2.** Table S1. Baseline demographic characteristics and laboratory data of the study cases with completed follow-up and those withdrawn from the study. Table S2. Multivariate Cox proportional hazard models for the prediction of kidney disease progression by different end-points among patients with coronary artery disease.
(RTF)
