# Supplementary material for: Progression of Kidney Disease in Non-Diabetic Patients with Coronary Artery Disease: Predictive Role of Circulating Matrix Metalloproteinase-2, -3, and -9
Source: PLoS One. 2013 Jul 26;8(7):e70132. doi: 10.1371/journal.pone.0070132 (PMC3724836; doi:10.1371/journal.pone.0070132)
Supplement: File S1 — Includes Table S1 and S2. Table S1. Baseline demographic characteristics and laboratory data of the study cases with completed follow-up and those withdrawn from the study. Table S2. Multivariate Cox proportional hazard models for the prediction of kidney disease progression by different end-points among patients with coronary artery disease. (RTF) [file pone.0070132.s001.rtf]

Supporting Information

Progression of Kidney Disease in Non-diabetic Patients with Coronary Artery Disease: Predictive Role of Circulating Matrix Metalloproteinase-2, -3, and -9

Ta-Wei Hsu1, Ko-Lin Kuo2, Szu-Chun Hung2, Po-Hsun Huang3,6, Jaw-Wen Chen3, and Der-Cherng Tarng4,5,6* 

1 Division of Nephrology, National Yang-Ming University Hospital, Yilan, Taiwan, 2 Division of Nephrology, Buddhist Tzu Chi Hospital Taipei Branch, Taipei, Taiwan; 3 Division of Cardiology, Taipei Veterans General Hospital, Taipei, Taiwan, 4 Division of Nephrology, Department of Medicine, Taipei Veterans General Hospital, Taipei, Taiwan; 5 Department and Institute of Physiology, National Yang-Ming University, Taipei, Taiwan, 6 Institute of Clinical Medicine, National Yang-Ming University, Taipei, Taiwan

* E-mail: dctarng@vghtpe.gov.tw


Table S1 Baseline demographic characteristics and laboratory data of the study cases
	Total Cases	Cases with completed follow-up	Cases withdrawn from the study		
Parameters	(n = 346)	(n = 251)	(n = 95)	P valuea	
Age (years)	65 ± 11	66 ± 11	64 ± 12	0.17	
Male gender (%)	83.5	84.1	82.1	0.65	
Hypertension (%)	62.7	64.2	57.9	0.32	
Smoking history (%)	28.6	26.4	35.7	0.13	
Body mass index (kg/m2)	25.1 ± 3.3	25.2 ± 3.4	24.8 ± 3.0	0.38	
Coronary artery statusb				0.36	
Normal coronary artery (%)	13.6	12.0	17.9		
Insignificant CAD (%)	36.1	37.8	31.6		
Significant CAD (%)	50.3	50.2	50.5		
Baseline eGFR (mL/min per 1.73 m2)	74.2 ± 16.0	74.1 ± 15.3	74.5 ± 17.8	0.83	
Fasting glucose (mg/dL)	96.8 ± 15.6	97.0 ± 15.8	96.0 ± 15.3	0.61	
Lipid profile					
Triglyceride (mg/dL)	125 ± 61	127 ± 64	120 ± 53	0.29	
Total cholesterol (mg/dL)	184 ± 34	185 ± 32	182 ± 37	0.47	
LDL-cholesterol (mg/dL)	117 ± 30	117 ± 29	116 ± 32	0.89	
HDL-cholesterol (mg/dL)	42 ± 12	 42 ± 11	42 ± 12	0.85	
Serum albumin (g/dL)	4.10 ± 0.34	4.11 ± 0.34	4.07 ± 0.33	0.34	
Calcium (mg/dL)	8.9 ± 0.5	8.9 ± 0.5	8.9 ± 0.5	0.91	
Phosphate (mg/dL)	3.4 ± 0.6	3.3 ± 0.6	3.5 ± 0.6	0.80	
Uric acid (mg/dL)	6.9 ± 1.9	6.9 ± 2.0	6.9 ± 1.8	0.88	
High-sensitive CRP (mg/L)	0.86 [0.45 to 2.43]	0.83 [0.41 to 2.26]	1.00 [0.45 to 2.66]	0.51	
MMP-2 (ng/mL)	884 [769 to 1008]	865 [789 to 1007]	875 [742 to 1023]	0.68	
MMP-3 (ng/mL)	217 [141 to 275]	206 [136 to 271]	215 [155 to 283]	0.71	
MMP-9 (ng/mL)	43 [29 to 58]	45 [26 to 53]	42 [24 to 53]	0.92	
Medications at enrollment					
Antiplatelet agents (%)	83.2	82.1	86.3	0.35	
Nitrate (%)	34.7	37.8	27.4	0.08	
Calcium channel blockers					
DHP (%)	17.1	18.7	12.6	0.18	
Non-DHP (%)	30.4	28.7	27.8	0.81	
 blockers (%)	16.8	18.3	12.6	0.21	
ACEI and/or ARB use (%)	44.1	43.0	47.3	0.47	
Statins (%)	19.7	21.1	15.8	0.27	
aComparison between two groups of patients who did and did not complete the study by the student's t test, Pearson x2 test, or the Mann-Whitney U test, as appropriate.
bAtherosclerotic lesion <20% was denoted as normal coronary artery, lesion 20-50% of stenosis as insignificant CAD, and lesions more than 50% of stenosis as significant CAD.
Abbreviations: ACEI, angiotensin-converting enzyme inhibitors; ARB, angiotensin II receptor blockers; CAD, coronary artery disease; CRP, C-reactive protein; DHP, dihydropyridine; eGFR, estimated glomerular filtration rate; HDL, high density lipoprotein; LDL, low density lipoprotein; MMP, matrix metalloproteinase.


Table S2 Multivariate Cox proportional hazard models for the prediction of kidney disease progression by different end-points among patients with coronary artery disease 
End-points	  Hazard Ratio	95% CI	P value	
eGFR decline >20% from baseline				
eGFR: per 10 mL/min per 1.73 m2 increase	0.83	[0.66 to 0.82]	<0.01	
MMP-2 ≥ 861 ng/mL	1.66	[1.21 to 2.04]	0.02	
MMP-3 ≥ 227 ng/mL	1.58	[1.01 to 2.51]	0.03	
MMP-9 ≥ 49 ng/mL	3.24	[1.80 to 5.43]	<0.01	
Final eGFR <60 mL/min per 1.73 m2				
eGFR: per 10 mL/min per 1.73 m2 increase	0.55	[0.43 to 0.68]	<0.01	
MMP-2 ≥ 861 ng/mL	1.73	[0.96 to 3.14]	0.07	
MMP-3 ≥ 227 ng/mL	1.02	[0.99 to 1.04]	0.09	
MMP-9 ≥ 49 ng/mL	1.90	[1.06 to 3.41]	0.03	
Medians of baseline plasma MMP-2, MMP-3 and MMP-9 levels were 861 ng/mL, 227 ng/mL, and 49 ng/mL, respectively.
CI, confidence interval; eGFR, estimated glomerular filtration rate; MMP, matrix metalloproteinase.
